# Tissue-Specific Methylation of Human Insulin Gene and PCR Assay for Monitoring Beta Cell Death

**DOI:** 10.1371/journal.pone.0094591

**Published:** 2014-04-10

**Authors:** Mohamed I. Husseiny, Alexander Kaye, Emily Zebadua, Fouad Kandeel, Kevin Ferreri

**Affiliations:** 1 Department of Diabetes and Metabolic Diseases Research, Beckman Research Institute of City of Hope, Duarte, California, United States of America; 2 Faculty of Pharmacy, Zagazig University, Zagazig, Egypt; La Jolla Institute for Allergy and Immunology, United States of America

## Abstract

The onset of metabolic dysregulation in type 1 diabetes (T1D) occurs after autoimmune destruction of the majority of pancreatic insulin-producing beta cells. We previously demonstrated that the DNA encoding the insulin gene is uniquely unmethylated in these cells and then developed a methylation-specific PCR (MSP) assay to identify circulating beta cell DNA in streptozotocin-treated mice prior to the rise in blood glucose. The current study extends to autoimmune non-obese diabetic (NOD) mice and humans, showing in NOD mice that beta cell death occurs six weeks before the rise in blood sugar and coincides with the onset of islet infiltration by immune cells, demonstrating the utility of MSP for monitoring T1D. We previously reported unique patterns of methylation of the human insulin gene, and now extend this to other human tissues. The methylation patterns of the human insulin promoter, intron 1, exon 2, and intron 2 were determined in several normal human tissues. Similar to our previous report, the human insulin promoter was unmethylated in beta cells, but methylated in all other tissues tested. In contrast, intron 1, exon 2 and intron 2 did not exhibit any tissue-specific DNA methylation pattern. Subsequently, a human MSP assay was developed based on the methylation pattern of the insulin promoter and human islet DNA was successfully detected in circulation of T1D patients after islet transplantation therapy. Signal levels of normal controls and pre-transplant samples were shown to be similar, but increased dramatically after islet transplantation. In plasma the signal declines with time but in whole blood remains elevated for at least two weeks, indicating that association of beta cell DNA with blood cells prolongs the signal. This assay provides an effective method to monitor beta cell destruction in early T1D and in islet transplantation therapy.

## Introduction

Currently it is estimated that there are more than 347 million diabetics worldwide [1] of whom 5%–10% have type 1 diabetes (T1D). Furthermore, the incidence of T1D is increasing dramatically [2]. A recent report from Europe estimates that new cases of T1D in children up to 14 years old are increasing 3%–4% annually [3]. T1D is a chronic autoimmune disease [4]–[7] characterized by the silent destruction of insulin-producing beta cells. The autoimmune process involves cytotoxic T cells [8], anti-islet antibodies [9], and antigen presenting cells [10] and can occur over several years prior to the loss of metabolic control [11], [12]. Beta cell destruction initially results in an impaired insulin response [13], [14], but persistent hyperglycemia follows after loss of about 65% of the beta cell mass [15]. Early detection of beta cell death would allow for earlier interventions to prevent significant loss of residual beta cell mass and function. We and others have recently developed methods for monitoring beta cell death by specific detection of beta cell-derived unmethylated DNA in circulation before the onset of diabetes [16]–[18]. This approach is based on the observation that the DNA encoding portions of the insulin gene in mice (*Ins2*) and humans (*INS*) is tissue-specifically unmethylated in insulin-producing beta cells [19], and that this DNA is released into circulation when beta cells are lost during the progression of the disease. To monitor beta cell death, we developed a highly sensitive quantitative methylation-specific PCR (qMSP) assay to detect the circulating beta cell DNA and demonstrated that this signal appears prior to hyperglycemia in a chemically-induced diabetic mouse model [16].

Here we extend these results to an autoimmune mouse model of T1D by showing that the beta cell-specific DNA appears in circulation well before the appearance of diabetes in these animals. In applying this assay to humans, we then mapped the methylation patterns of the human insulin gene in several tissues and demonstrated that the promoter, but not intron 1, exon 2, and intron 2, exhibited tissue-specific methylation. Based on this information a modified MSP assay using a two-step nested approach was developed and used to monitor circulating beta cell DNA following clinical islet transplantation therapy.

## Materials and Methods

### Animals and Ethics Statement

The study was approved by the City of Hope Institutional Animal Care and Use Committee (IACUC# 07031). The animal care facility at the City of Hope has been approved by the NIH, registered with the U.S. Department of Agriculture, and fully accredited by the Association for Assessment and Accreditation of Laboratory Animal Care International (AAALAC). All surgery was performed under isoflurane anesthesia, and all efforts were made to minimize suffering. The experiments were designed to utilize the minimum number of animals required to obtain valid results. Eight week old female NOD/ShiLtJ mice were obtained from The Jackson Laboratory (Bar Harbor, Maine, USA) and maintained under specific pathogen-free conditions. Every two weeks, approximately 200 μl blood was collected from each mouse for DNA purification and bisulfite conversion then used as a template for qMSP assay. Blood glucose was measured every two weeks with One Touch Ultra glucometer (LifeScan, Milpitas, CA, USA) and the animals were considered to be diabetic when levels remained above 200 mg/dL.

### Insulitis score

Every two weeks the pancreata were removed from 5 randomly chosen mice. The tissues were fixed in formalin and stained with hematoxylin and eosin (H&E). An insulitis score for each islet was determined (0 =  no insulitis; 1 =  peri-insulitis; 2 =  mild insulitis with <50% islet area affected; 3 =  invasive insulitis with.>50% islet area affected). Between 21 and 59 islets were scored from 5 mice at each time interval.

### Human Subjects

Human tissues and islet cell isolation at the City of Hope National Medical Center was performed with the approval of the City of Hope Institutional Review Board (IRB# 01083, 08078, 08079, and 05058). Written informed consent from the donors for research use of tissue in this study was obtained prior to acquisition of the tissues. Beta cells were enriched as previously described [19]. Briefly, islets were dissociated with TrypLE (Invitrogen, Carlsbad, CA) and stained with Newport Green (Invitrogen, Carlsbad, CA) before enriching by fluorescent-activated cell sorting. Normal human tissues such as liver, breast, colon, kidney, lung, spleen, and stomach were obtained from the Pathology Core at City of Hope. Blood samples were collected from normal healthy controls and from patients before and after islet transplantation.

### Isolation of genomic DNA

Genomic DNA was obtained from human tissues using NucleoSpin Tissue (Clontech, Mountain View, CA). In case of plasma obtained from blood of transplant recipient, gDNA was purified using QIAamp MinElute Virus Spin Kit and gDNA obtained from blood of transplant recipient was purified using QIAamp Blood Medi Kit (QIAGEN, Valencia, CA).

### PCR cloning of a fragment from human insulin gene

Primers H-INS-pro-For and H-INS-exon2-Rev (Table S1) were used to amplify a 900 bp fragment from human gDNA containing the promoter, intron 1, exon 2, and intron 2 of the human insulin gene (*INS*). The PCR product was cloned into pCR2.1-TOPO plasmid vector (Invitrogen, Carlsbad, CA) and used for development of the assay. The cloned sequence was confirmed by the DNA Sequencing/Solexa Core at the Beckman Research Institute of City of Hope using M13F and M13R primers.

### Bisulfite genomic sequencing

Nucleotide sequence of the human *INS* (GeneID: 3630) gene was obtained from Genbank and the potential methylation sites (i.e. CG dinucleotides) were identified. Genomic DNA was isolated from various tissues as described above and were treated with EZ DNA methylation-gold kit (Zymo Research, Orange, CA) according to the manufacturer's recommendation. The human *INS* gene was then amplified with pairs of gene-specific primers for promoter and exon 2 (Table S1) in a mixture containing 100 ng bisulfite modified DNA as a template and DNA Hot Star-Taq polymerase (QIAGEN, Valencia, CA). Each PCR fragment was TA cloned into pCR2.1-TOPO vector and sequenced as described above. Each pattern resulted from 20–61 clones of *INS* promoter or 5–35 clones of *INS* exon 2 (Figure S2) obtained from 6 different individuals for blood tissue and 3 individuals from other tissues as indicated in (Table S2). Statistics of each CpG site were done using the QUMA computer program (http://quma.cdb.riken.jp/) which performs a Fisher exact test.

### Nested methylation-specific PCR (MSP)

Quantitative PCR was performed with a 7500 Real-time PCR instrument (Applied Biosystems, Foster City, CA). In First-Step PCR, each reaction contained 20–30 ng of bisulfite-treated DNA, 12.5 μl QuantiTect SYBR Green PCR (QIAGEN, Valencia, CA) and 500 nM each forward and reverse primer (Table S1) in a total volume of 25 μl. Thermal cycling was initiated with an enzyme activation step of 15 min at 95°C, followed by 15 cycles of 95°C for 15 s, 60°C for 30 s, and, 72°C for 30 s. The PCR products were purified using QIAquick PCR Purification Kit (QIAGEN, Valencia, CA). In Second-Step PCR, the products from the first reaction were used as template for a qPCR with nested primers (Table S1). The reactions were initiated with for 15 min at 95°C, followed by 40 cycles of 95°C for 15 s, 57°C for 30 s, and, 72°C for 30 s. The quantification cycle (C_q_) was determined for each reaction with methylation-specific primers (MSP) and bisulfite-specific primers (BSP) and the ratio of unmethylated to total amplifiable bisulfite-treated DNA was calculated using the Relative Unmethylation Ratio (RUR) as previously described by Husseiny et al. [16] as Relative Expression Ratio (RER). The second-step reaction C_q_ values were between 15 and 40. Negative controls without DNA did not yield products in the first-step reaction.

### Statistical analysis

Statistical significance between samples was tested with a two-tailed Student's t-test for unpaired values or two-way analysis of variance (ANOVA) between human islets and other tissues (colon and blood) using GraphPad Prism 6 software. Statistical significance was defined as a p-value of <0.05, <0.01, and <0.001. Statistical analysis of DNA methylation was done using QUMA (http://quma.cdb.riken.jp/) which performs a Fisher exact test on the methylation status of individual CpG sites using p<0.1, and p<0.01. Data are expressed as mean ± SEM.

## Results

### Evaluation of qMSP for detection of circulating beta cell-specific DNA in an autoimmune mouse model

**Figure 1:**
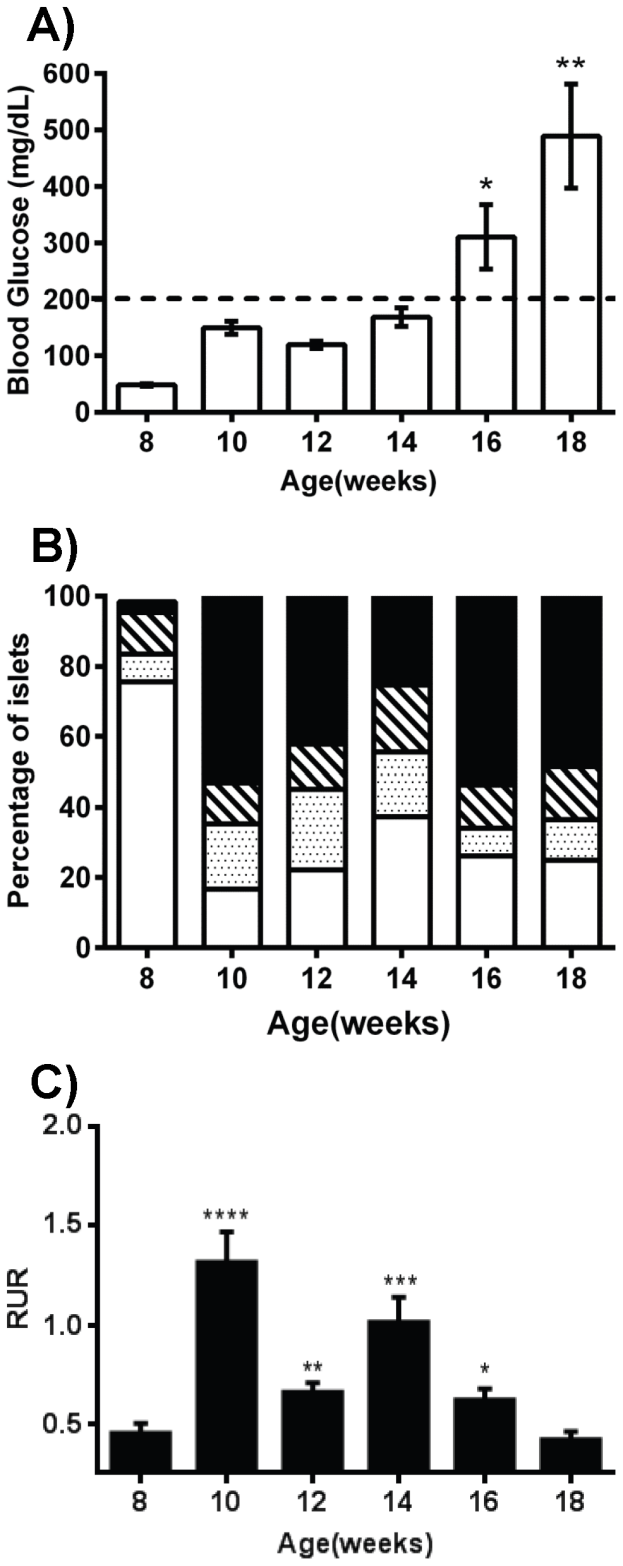
Detection of circulating beta cell DNA in NOD mice using qMSP assay. A) Blood glucose levels in NOD mice measured every two weeks (n = 5) showing a significant rise in mean blood glucose levels after 16 weeks. The dashed line (200 mg/dL) indicates the hyperglycemic threshold. B) Pancreatic sections of the indicated groups (n = 5) were stained with H&E and the degree of insulitis was scored: no insulitis (white), peri-insulitis (dotted), mild insulitis (hatched), and invasive insulitis (black). C) In parallel, circulating beta cell DNA was measured by qMSP in each mouse group (n = 5) at designated time points. Fold changes in unmethylation are quantified by calculation of the Relative Unmethylation Ration (RUR) for each sample (see Experimental Design and Methods). The data display the mean ± standard error mean (SEM) of three independent measurements. The statistical significance was calculated by unpaired *t* tests compared with week 8 values and indicated by asterisks (*, p<0.05; **, p<0.01; ***, p<0.001; ****, p<0.0001).

To assess application of qMSP assays to autoimmune-mediated loss of beta cells, the previously developed assay [16] was used to monitor the development of diabetes in the non-obese diabetic (NOD) autoimmune mouse model which shares many similarities with T1D in humans. As can be seen, the mice become significantly hyperglycemic (>200 mg glucose/dL) at weeks 16 and 18 (p = 0.01 and p = 0.004, respectively) (Figure 1A). However, the lymphocyte infiltration of the islets (insulitis, Figure S1) is mildly present even at 8 weeks and shows a significant increase by week 10 and remains high through weeks 12, 14, 16 and 18 (p = 0.0007, p = 0.01, p = 0.009, p = 0.001, and p = 0.0007, respectively compared with week 8) (Figure 1B). Concomitantly, there is a significant rise in circulating unmethylated beta cell-specific DNA starting at week 10 (Figure 1C) which remains elevated at weeks 12, 14, and 16 (p<0.0001, p = 0.0053, p = 0.0008, and p = 0.04, respectively compared with week 8) until it drops to baseline levels at week 18. These results demonstrate that even in a spontaneous diabetic model that the MSP methodology is capable of detecting beta cell death at the onset of insulitis and six weeks prior to the rise in blood sugar.

### Methylation pattern of the human insulin gene

We previously reported that the promoter of the human insulin gene (*INS*) contains nine potential methylation (CpG dinucleotide) sites that are located at positions −357, −345, −234, −206, −180, −135, −102, −69 and −19 bp relative to the transcription start site (TSS) and that these sites are predominantly unmethylated in pancreatic beta cells yet methylated in other pancreatic cells [19]. However, monitoring beta cell death by qMSP critically depends on the specificity of the beta cell methylation pattern, and so a broader examination of the tissue-dependent methylation of the human insulin gene was performed.

**Figure 2:**
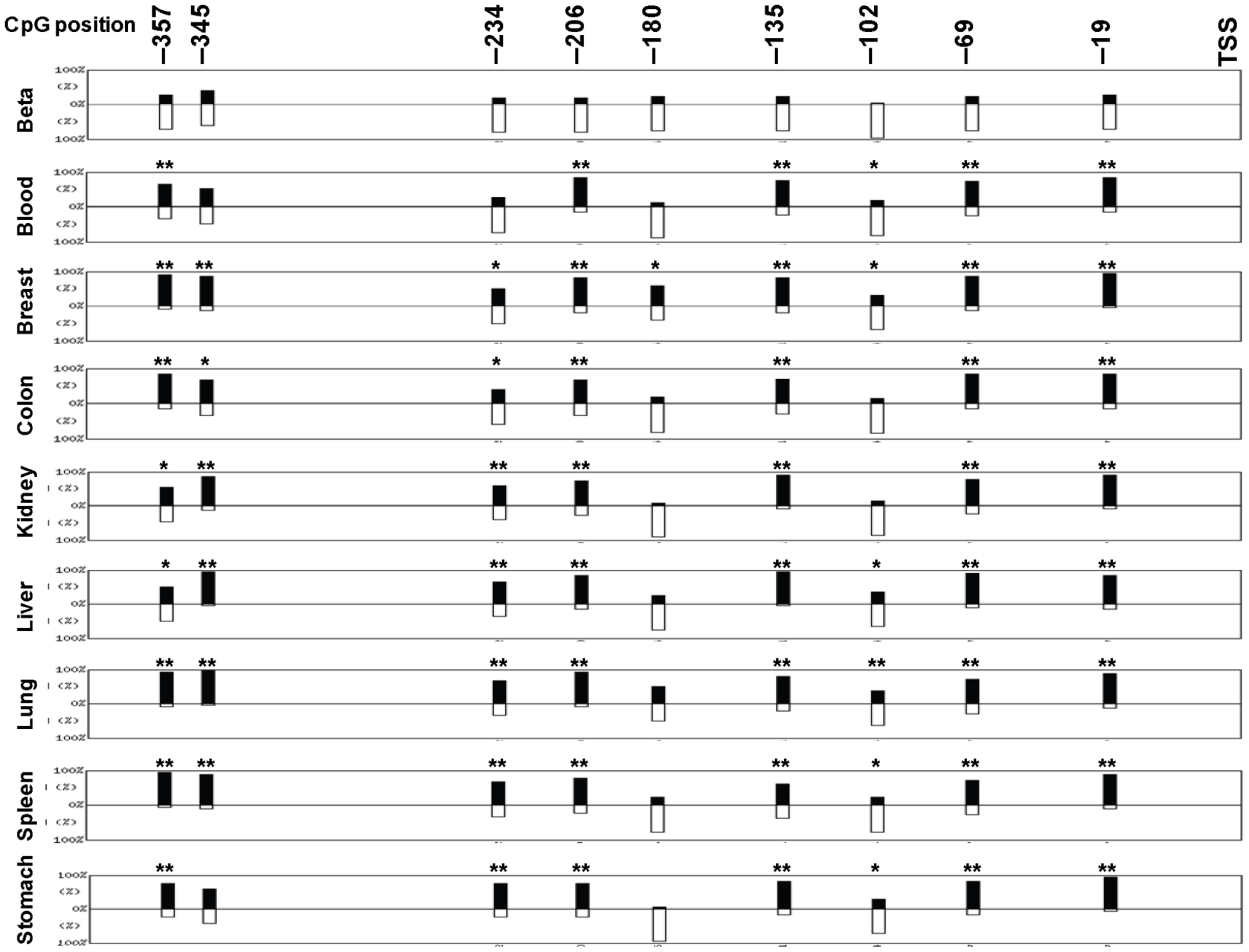
Tissue-specific methylation of the human *INS* promoter. Genomic DNA samples obtained from human blood, breast, colon, kidney, liver, lung, spleen, stomach, and human beta cells (‘Islet cell fraction’≈70% beta cells) were analyzed for methylation of the *INS* promoter. The positions of the nine CpG sites relative to the transcription starting site (TSS) are indicated. The bars display the position and the percentage of unmethylation (white bars) to methylation (black bars) for each CpG. Each pattern results from 20 to 61 clones and obtained from 6 individuals for blood and 3 individuals for other tissues. Statistics were done using the QUMA computer program and Fisher exact test comparing each site with the same site in beta cells. The statistical significance is indicated by asterisks (*, p<0.1; **, p<0.01).

**Figure 3:**
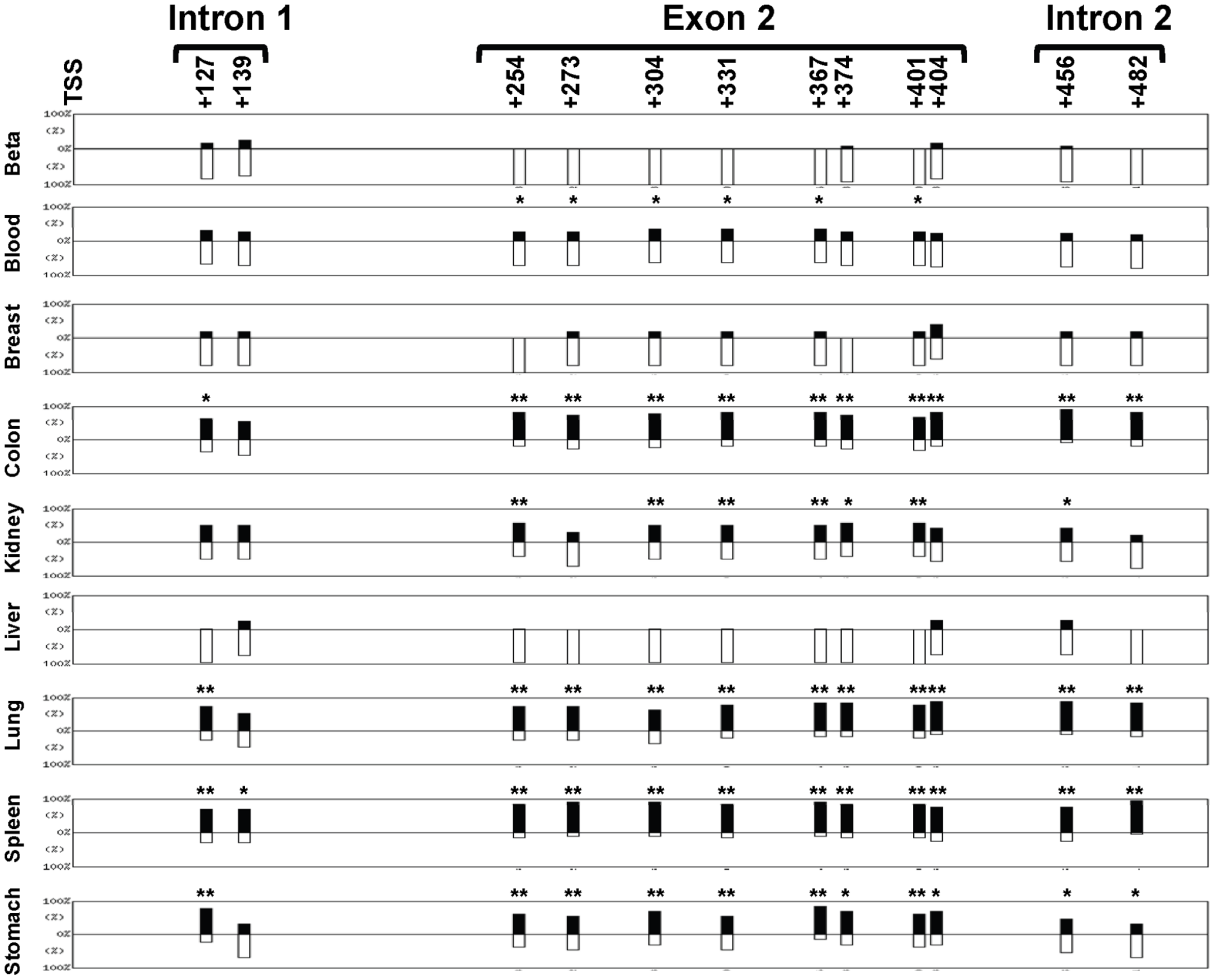
Tissue methylation pattern of the human *INS* exon 2. The human tissues shown in Figure 2 were analyzed for methylation of *INS* exon 2 (8 CpG), intron 1 (2 CpG) and intron 2 (2 CpG). The positions of the twelve CpG sites relative to the transcription starting site (TSS) are indicated. The bars display the position and percentage of unmethylation (white bars) to methylation (black bars) for each CpG. Each pattern results from 5 to 35 clones obtained from 5 individuals blood, 2 individuals beta cells and breast, and 3 individuals for the other tissues. Statistics were done using the QUMA computer program and Fisher exact test comparing each site with the same site in beta cells. The statistical significance is indicated by asterisks (*, p<0.1; **, p<0.01).

Genomic DNA from nine different tissues including an enriched beta cell fraction were subjected to bisulfite sequencing of the *INS* gene to map their respective CpG methylation patterns (Figures 2 and 3). Between 2 and 6 different donors were used for each tissue and between 5 to 61 clones were sequenced for each tissue (Table S2). As was previously observed, analysis of the bisulfite sequencing data revealed that most of the CpG sites in the *INS* promoter are uniquely unmethylated in pancreatic beta cells but predominantly methylated in other tissues (Figure 2). However, it is interesting to note that not all of the sites exhibit the same degree of tissue-dependent methylation. The CpGs at −102, −180, −234, and −345 were substantially unmethylated in all the tissues examined and were not substantially different from beta cells (Figure 2). Conversely, sites −19, −69, −135, −206, and −357 were unmethylated in beta cells but not other tissues, and display a significant tissue-specificity. These results demonstrated that only certain sites in the human *INS* promoter exhibit a tissue-specific DNA methylation pattern and therefore only these sites can differentiate between beta cells and other tissues.

To determine whether other regions of the human insulin gene are also preferentially unmethylated, exon 2 and the surrounding regions were bisulfite sequenced. As shown in Figure 3 there are eight CpG sites in the exon 2 located at positions +254, +273, +304, +331, +367, +374, +401, and +404 bp relative to the TSS. Two other sites in intron 1 (+127 and +139) and two sites in intron 2 (+456 and +482) were also examined. As in the promoter, these sites are mostly unmethylated in beta cells and variably methylated in colon, kidney, lung, spleen and stomach (Figures 3 and S2). However, intron 1, exon 2 and intron 2 were also found to be predominantly unmethylated in blood, breast and liver cells. Therefore, the human *INS* intron 1, exon 2, and intron 2 regions were not appropriate for targeting with the qMSP assay since it does not exhibit a beta cell-specific pattern, especially in blood, the major background signal of the assay.

### Quantitative methylation-specific PCR

**Figure 4:**
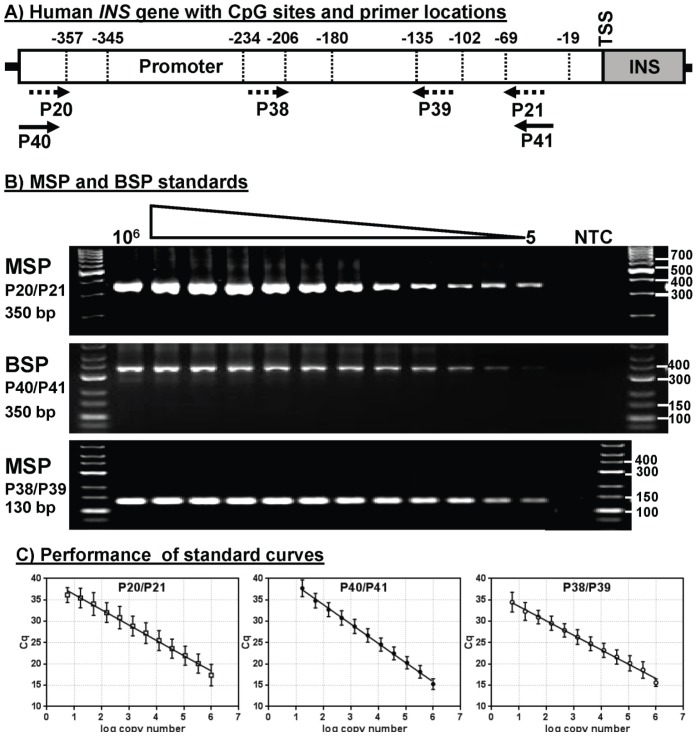
Primer selection and analytical performance of methylation-specific PCR. A) Schematic illustration of the human *INS* gene promoter region showing the position of the nine CpG sites. Solid arrows represent the bisulfite-specific primers (BSPs) that amplify both methylated and unmethylated DNA. Dashed arrows represent methylation-specific primers (MSPs) that amplify unmethylated DNA only. B) Unmethylated plasmid was serially diluted after bisulfite conversion and analyzed by qMSP using selected primer sets. Agarose gel electrophoresis of MSP reactions showing the size of the PCR products. C) Graphs of real-time SYBR Green PCR data showing linearity of C_q_ versus log copy number of unmethylated plasmid (averages and standard deviation (SD)) from 5 to 10^6^ copies.

To monitor circulating beta cell DNA in human samples, a qMSP was developed based on the results of the tissue-specific methylation mapping. The design of the human assay was similar to the assay developed for mice [16] except that there were two more differentially methylated sites (three in mice versus five in humans). This made it possible to utilize a nested PCR technique that would interrogate additional methylation sites while possibly improving both the specificity and sensitivity of the assay. Primers were designed to recognize only unmethylated DNA as found in beta cells (Figure 4A). Primers P20 and P21 are methylation-specific primers (Figure 4A, dashed arrows) targeting CpGs at −357 and −69, respectively, and together produce a product of 350 bp (Figure 4B). Primers P38 and P39 target CpGs −206 and −135, respectively, and produce a product of 130 bp (Figure 4B). In addition, two primers, P40 and P41, target the regions just upstream and downstream of P20 and P21, respectively, and were not aligned with any CpG site (Figure 4A, solid arrows). P40 and P41 are bisulfite-specific primers (BSP) and amplify a 350 bp product from both methylated and unmethylated DNA, and therefore provide a measure of total amplifiable insulin gene promoter sequences (Figure 4B). These primer sets were evaluated using serial dilutions of the cloned unmethylated insulin gene as a template. As shown, each MSP and BSP primer set exhibited dose dependent amplification ranging from 10^6^ copies to as few as 5 copies of the unmethylated sequences (Figure 4B).

Quantitative analysis of the standard curves shows that the BSP and MSP assays were linear over a 10^5^-fold range of template concentrations (Figure 4C). For nested PCR, the two MSP assays were applied sequentially, i.e. amplification with P20/P21 followed by P38/P39.Variation across the nested MSP curve ranged from 2.83% to 6.58% (Table 1). Furthermore, the standard curve parameters (Table 2) were highly reproducible for both nested qMSP (efficiency = 85.19% ±1.37 SD, slope = −3.737±0.05 SD, R^2^ = 0.979±0.004 SD; n = 5 experiments) and qBSP (efficiency = 80.19%±6.35 SD, slope = −3.925±0.23 SD, R^2^ = 0.982±0.016 SD; n = 5 experiments). The ability of the primer sets to distinguish methylated versus unmethylated templates was determined by PCR using increasing concentrations of the bisulfite-treated methylated or unmethylated plasmids. The qMSP assay readily distinguishes unmethylated and methylated plasmid at 10^4^ and 10 copies showing considerably higher signal (lower C_q_) and a detection limit of less than 10 copies of unmethylated sequences (Figure S3A). In contrast the BSP assay shows the same C_q_ values for both unmethylated and methylated sequences at 10^4^ and 10 copies (Figure S3B).

**Table 1:** Statistical variation of qMSP standard curve.

Log Copy Number	Average C_q_	±SD	%CV
1.7	28.22	0.80	2.83
2.2	25.79	0.92	3.57
2.7	24.29	0.75	3.09
3.1	23.01	0.79	3.44
3.6	21.35	0.68	3.21
4.1	20.07	0.77	3.86
4.6	17.89	.0.68	3.84
5.0	14.58	0.83	5.68
5.5	14.36	0.48	3.35
6.0	10.03	0.66	6.58

C_q_ is the average of n = 5; SD  =  standard deviation, %CV  =  percent coefficient of variation [(SD/C_q_ average) ×100].

**Table 2:** The amplification efficiency of qMSP and qBSP standard curves.

PCR type (Primer set)	Efficiency % ±SD	Slope ±SD	R^2^±SD
**Nested MSP (P38/P39)**	85.19±1.37	−3.737±0.05	0.979±0.004
**BSP (P40/P41)**	80.19±6.35	−43.925±0.23	0.982±0.016

MSP  =  methylation-specific PCR, BSP  =  bisulfite-specific PCR, Slope  =  slope of the standard curve, R^2^ =  the square of the correlation coefficient of the standard curve.

**Figure 5:**
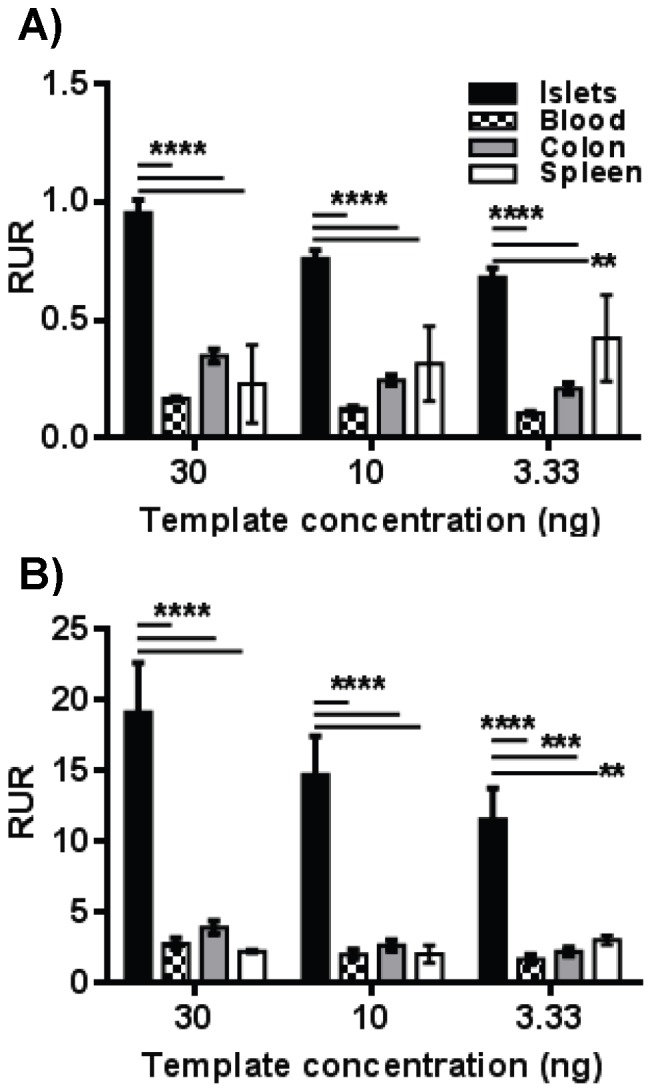
Beta cell specificity of MSP. Serial dilutions of the bisulfite-converted gDNA obtained from human islets, blood, spleen and colon were used as a template for nested PCR using either BSP (A) or MSP (B) in the first-step reaction. The products were used as a template for the second-step MSP reaction. The data display the mean ± SEM of the Relative Unmethylation Ratio (RUR). The cloned *INS* promoter was used for normalization and standardization of the results. Statistically significant differences at each DNA concentration between islets and other tissues were calculated using two way ANOVA and the significance level indicated by asterisks (****, p<0.0001; ***, p<0.001; **, p<0.01).

These primers sets were then tested for specificity and sensitivity employing serially diluted bisulfite converted gDNA from human islets, blood, spleen, and colon as templates for qMSP. Fold changes in unmethylation were calculated by the Relative Unmethylation Ratio (RUR) for each sample [16] in which the level of beta cell DNA (qMSP) was normalized for total amplifiable sequences (qBSP). To assess the effect of targeting 2 versus 4 CpG sites, the nested reaction using P38/P39 was preceded by a first reaction using either BSP primers (P40/P41) to target a total of 2 sites or MSP primers (P20/P21) to target 4 sites. The assay interrogating 2 CpG sites exhibited a highly significant specificity for islets over blood and colon (Figure 5A). However, the assay targeting 4 sites showed a greater difference in signal between islets and other tissues (two-way ANOVA between samples, p<0.0001), indicating that the specificity of the assay was increased by increasing the number of CpG sites interrogated in the assay (Figure 5B). Amplification curves and melting curves for these assays is shown in Figure S4.

### Quantitative MSP for monitoring circulating beta cell DNA in islet transplant patients

**Figure 6:**
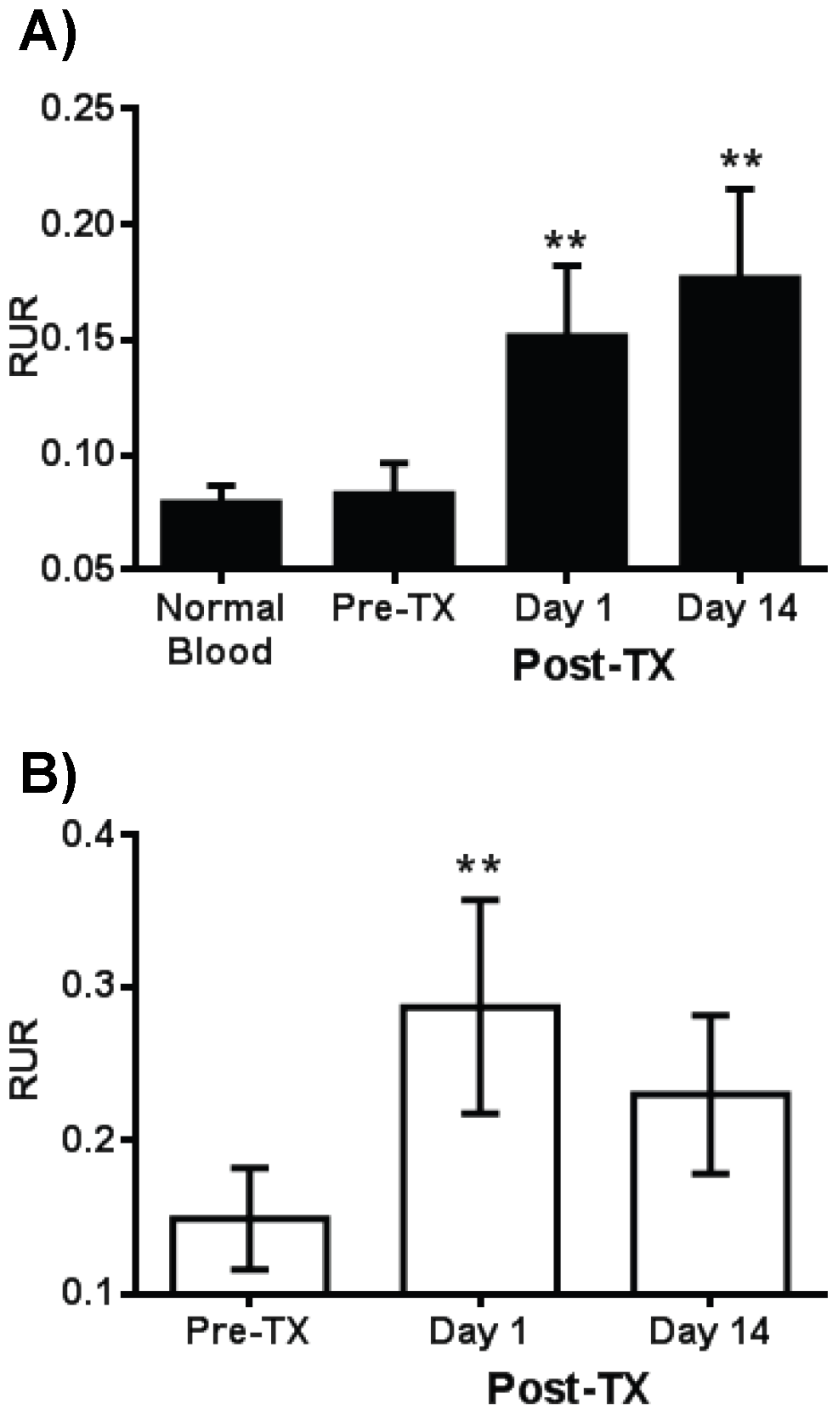
Quantitative MSP for monitoring beta cells in islet transplant patients. (A) Blood samples were collected from 6 normal healthy individuals and pre- and post- transplantation (at days 1 and 14 post-TX) from 6 islet transplant patients. Genomic DNA from the samples was bisulfite-converted and used for nested qMSP and BSP assays. The data display the mean ± SEM of the Relative Unmethylation Ratio (RUR) calculations. (B) Plasma samples were prepared from the islet recipient blood samples and analyzed as in A. The statistical significance was calculated with the Wilcoxon test to compare RUR of samples after transplant with that before transplant and significance level indicated by asterisks (*, p<0.05; **, p<0.01).

The application of the qMSP assay to human studies was assessed using blood samples from clinical islet transplant patients. The subjects (age 47±6 years) received an average of 4421±162 human islet equivalents/kg resulting in a 77%±5% decline in insulin requirements. Blood samples were obtained from islet recipients (n = 6) prior to transplantation (TX) and on post-transplant days 1 and 14. These were compared with blood from healthy donors (n = 6) using the qMSP assay. Plasma fractions were also prepared and compared with the results of whole blood to determine whether plasma was a better starting point for the assay. Prior to transplantation, there was no significant difference in the qMSP signal between the patient samples and normal controls (Figure 6A; Mann-Whitney U test; p = 0.92). However, the qMSP signal rose significantly the day after islet transplantation (Wilcoxon test; p = 0.005) and remained elevated for at least fourteen days (Wilcoxon test; p = 0.004) in the whole blood samples (Figure 6A). In plasma samples, the signal also rose significantly on day 1 (Figure 6B; Wilcoxon test; p = 0.003), though in contrast to whole blood, fell again by day 14 (Wilcoxon test; p = 0.58). It is intriguing to note that the difference between these samples was the presence of cells, indicating that beta cell DNA is associated with cells in the blood and prolongs the qMSP signal. These results demonstrate that qMSP can be used to monitor beta cell DNA in human clinical samples and that the duration of the signal is longer in whole blood than in plasma.

## Discussion

We previously described the tissue-specific unmethylation of the mouse and human insulin genes in insulin-producing pancreatic beta cells [16], [19]. Using this distinctive difference we developed a qMSP assay to differentiate between circulating methylated and unmethylated DNA released into the blood upon beta cell death at the onset of T1D. The MSP assay was applied in the STZ-induced diabetic mouse model and a significant increase in circulating beta cell DNA was detected prior to the rise in blood glucose levels [16]. In this study we applied the qMSP assay to monitoring diabetes in the spontaneous diabetic NOD mouse model. Being an autoimmune model, the loss of beta cells is more likely to mimic the time course of human T1D than the catastrophic beta cell death due to STZ-treatment. Still, the qMSP assay was able to detect beta cell DNA in circulation six weeks prior to a rise in blood glucose levels. Importantly, the rise in qMSP signal coincided with increased insulitis at 10 weeks, indicating a correlation between the signal and the immune-mediated beta cell destruction associated with T1D, which also occurs in human T1D [20], [21].

The tissue-specific pattern of DNA methylation is important for development of a qMSP assay. This provides accurate identification of the cell source of circulating DNA, and therefore the underlying pathology associated with changes in the signal. We believe that careful mapping of the methylation pattern of the target gene in several tissues is critical to reliable application of this methodology to clinical diagnostics. In our previous report [19] we identified nine CpG sites in the human *INS* promoter, and found that the all of these were substantially unmethylated in islets but not other pancreatic cells, and that unmethylation was essential for insulin gene expression. In this study, comparisons with several other tissues confirmed that the unmethylation of the insulin promoter was specific to beta cells, but it was also found that not all CpG sites exhibited the same tissue-specificity. CpG sites at positions −19, −69, −135, −206, and −357 bp exhibited unique beta cell unmethylation, but CpG sites at −102, −180, −234 and −345 bp were not specific. These results demonstrate for the first time that though the methylation pattern of the human *INS* promoter is generally tissue-specific, some of the CpG sites are not appropriate for targeting in a diagnostic assay for T1D.

We previously found that the exon 2 region of the mouse *Ins2* gene also displayed tissue-specific methylation [16] similar to the promoter region. Consequently, we were surprised to find that the 8 CpG sites in human *INS* exon 2 failed to show tissue-specific differential methylation. Furthermore the 2 CpG sites in each of the human intron 1 and intron 2 did not exhibit tissue-specific differential methylation. It should be noted that our bisulfite sequencing results of *INS* exon 2 are in agreement with those of other recent reports showing differential methylation of CpG sites of this region between human beta cells and kidney [17], [22], which led those authors to develop a qMSP assay based on *INS* exon 2. However, an extensive examination of the tissue-specificity of this region reveals that the exon 2 region cannot be used to differentiate between DNA obtained from beta cells and from other tissues. It should also be noted that the analyses described in the other studies analyzed from 10 to 14 clones [17], [22], presumably from one individual for each tissue, whereas our analysis included 3 or more different individuals with at least 5 clones for most tissues (Table S2).The meaning of the similar methylation patterns in beta cells and these other tissues is unclear at this point, though some tissues, such as liver, have a clear developmental relationship with the pancreas [23]. However, from the view of assay development, these results, especially the lack of tissue-specific differences between blood and beta cells, demonstrate that the human *INS* exon 2 cannot be used for monitoring beta cell DNA in circulation by qMSP.

In this study, we were able to detect circulating beta cell DNA in human islet transplant recipients using qMSP starting at one day post-transplantation and continuing on through the next two weeks. These were long-term, type 1 diabetic patients with very low residual beta mass (stimulated C-peptide <0.6 ng/ml) prior to the procedure. Their pre-transplant qMSP signal level was identical to normal human controls, indicating a stable background signal in this assay that would allow for meaningful comparisons within patient data sets. This was partly due to the inclusion of intra- and inter-assay controls in the calculations as shown previously [16], but was also a result of the nested procedure that allowed for interrogation of up to four individual methylation sites and thus improved specificity of the assay (Figure 5). Another interesting aspect of this data was the difference in the signal persistence between the plasma fraction and whole blood, even though they were derived from the same samples. The implication we were left to make was that the presence of blood cells may be protecting the beta cell DNA in circulation in some unknown manner. Nevertheless, these results demonstrated the application of the qMSP assay to human samples, and we are currently using it to investigate the early stages of human T1D.

Several effective predictive biomarkers for progression to T1D have been described such as HLA [24] and other genotypes [25], autoantibodies [26], and autoreactive T-cell assays [27]–[30]. All of these markers appear to be associated with the autoimmune process itself and each can identify individuals at high risk for this disease. However, the target of the autoimmunity is the beta cells and the rising blood glucose is due to beta cell dysfunction and death. A recent study showed a correlation between autoantibody positivity and loss of beta cell function [31], but the we believe that a direct measure of beta cell death, such as the qMSP assay, will identify individuals not only at risk, but with actual ongoing disease prior to loss of metabolic control. This would allow for the functional screening of at risk individuals, monitoring of ongoing disease, as well as in the evaluation of treatment methods including islet cell transplantation.

In conclusion, the *INS* promoter in human beta cells exhibits a tissue-specific methylation pattern that can be used to distinguish beta cell DNA from DNA of other tissues. Interestingly, not all methylation of CpG sites in INS promoter of human beta cells are tissue-specific, so choosing the CpG sites was critical for development of MSP-dependent assay. We have also shown for the first time that intron 1, exon 2, and intron 2 of the human *INS* gene are not differentially methylated and are not suitable for targeting in tissue-specific diagnostics. A methylation-specific PCR assay was developed from the methylation data and is both sensitive and specific enough for the detection of circulating DNA released as a consequence of beta cell death, and application of this approach was demonstrated in a mouse model of T1D. The sensitivity and specificity of these assays will allow for an effective method to monitor beta cell destruction in mice and humans both in early T1D and in islet transplantation therapy.

## Supporting Information

Figure S1 **Insulitis progression in NOD mice.** Representative pancreatic paraffin sections of NOD mice at different ages were stained with hematoxylin and eosin (H&E), showing different stages of insulitis. Islets were observed using an Olympus IX51 fluorescent microscope equipped with an Infinity-2 camera (Olympus America, Melville, NY). Pictures were captured using Infinity Analyze acquisition 5.0 software (Lumenera Corporation, Ottawa, Canada.
(TIFF)

Figure S2 **Methylation patterns of human** ***INS*** **exon2 obtained from different individuals.** The human *INS* gene exon 2 was amplified with pairs of gene-specific primers for exon 2 (Table S1) using bisulfite-converted gDNA isolated from various tissues and different donors (brackets). Each PCR fragment was TA cloned into pCR2.1-TOPO vector and sequenced as described in Materials and Methods. PCR sequences were aligned with the expected sequence using the QUMA computer program (http://quma.cdb.riken.jp/). (A) Methylation pattern for beta cells resulted from 12 clones obtained from 2 individuals, (B) liver tissues resulted from 35 clones from 3 individuals, (C) kidney tissues resulted from14 clones from 3 individuals, and (D) blood tissues resulted from 27 clones from 5 individuals as indicated in (Table S2).
(TIFF)

Figure S3 **Differentiation between methylated and unmethylated sequences.** The cloned human *INS* fragment in the pCR2.1 plasmid was methylated using M.SssI CpG methyltransferase, which methylates all cytosine residues within the double-stranded dinucleotide recognition sequence CpG. (A) Methylation-specific PCR (MSP) using primers P20/P21. (B) Bisulfite-specific PCR (BSP) using primers P40/P41. Amplification plots (upper) and melting curves (lower). The qMSP assay for 10^4^ copies of the unmethylated sequence (red line) shows lower C_q_ values than 10^4^ copies of the methylated sequence (blue line), and qMSP can detect 10 copies for unmethylated but not methylated sequence. In contrast the BSP assay shows the same C_q_ values for both unmethylated and methylated sequences at 10^4^ and 10 copies.
(TIFF)

Figure S4 **Melting curves from the 2^nd^ step nested PCR reactions specific for beta cells.** Bisulfite-converted gDNA obtained from human islets, blood and colon were used as a template for nested PCR. (A) First step MSP reaction using primers (P20/P21) followed by second step MSP reaction using primers P38/P39. (B) First step BSP reaction using primers (P40/P41) followed by second step BSP reaction using primers P40/P41. Amplification plots (upper) and melting curves (lower). The qMSP assay for human islets (green line) shows lower C_q_ values than human colon (blue line) and blood (red line) In contrast the qBSP assay shows the same C_q_ values for all tissues.
(TIFF)

Table S1 **Oligonucleotides used in this study.**
(DOCX)

Table S2 **Mapping of human insulin promoter and exon 2 regions.**
(DOCX)
