# Supplementary figures and images for: Tissue-Specific Methylation of Human Insulin Gene and PCR Assay for Monitoring Beta Cell Death
Source: PLoS One. 2014 Apr 10;9(4):e94591. doi: 10.1371/journal.pone.0094591 (PMC3983232; doi:10.1371/journal.pone.0094591)

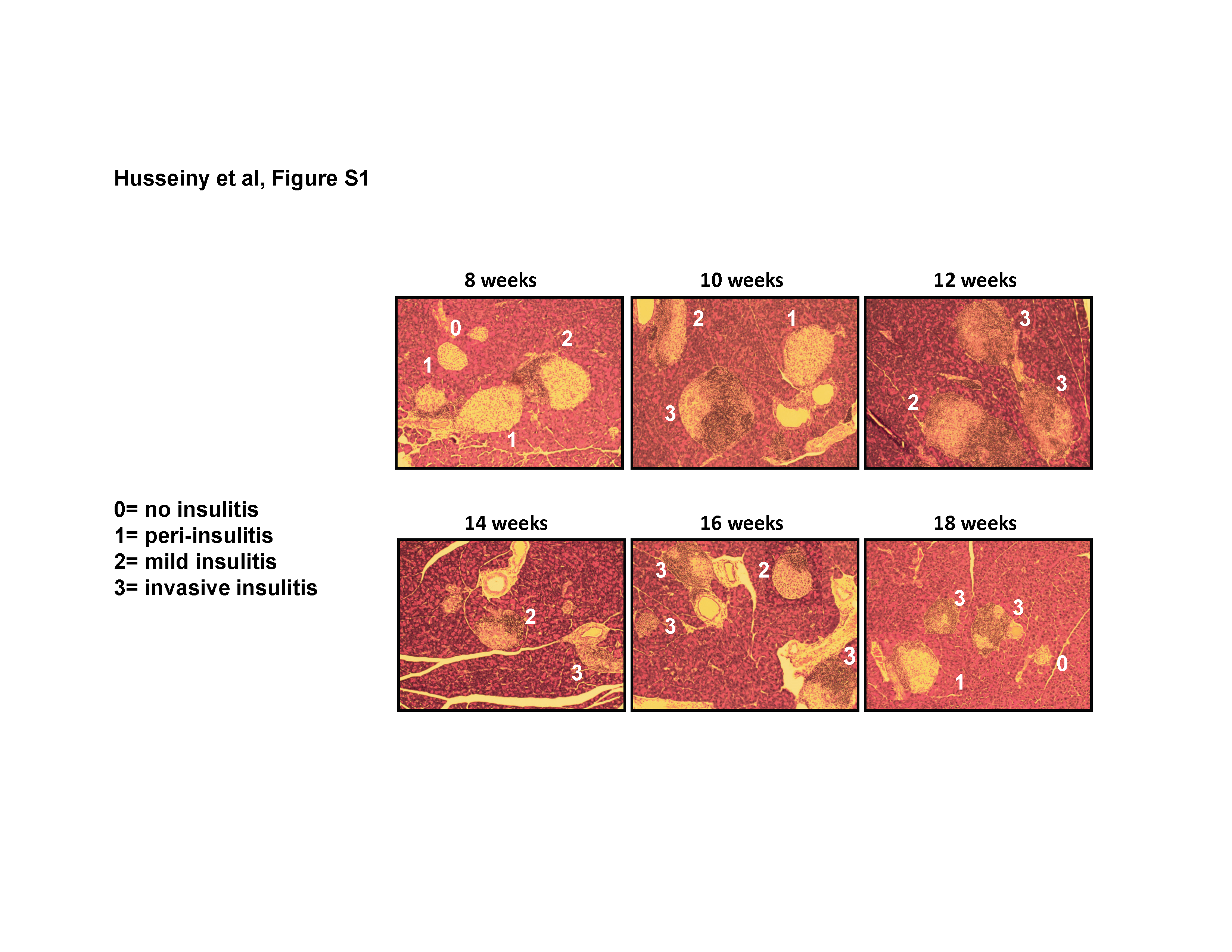

Supplement: Figure S1 — Insulitis progression in NOD mice. Representative pancreatic paraffin sections of NOD mice at different ages were stained with hematoxylin and eosin (H&E), showing different stages of insulitis. Islets were observed using an Olympus IX51 fluorescent microscope equipped with an Infinity-2 camera (Olympus America, Melville, NY). Pictures were captured using Infinity Analyze acquisition 5.0 software (Lumenera Corporation, Ottawa, Canada. (TIFF) [file pone.0094591.s001.tiff]

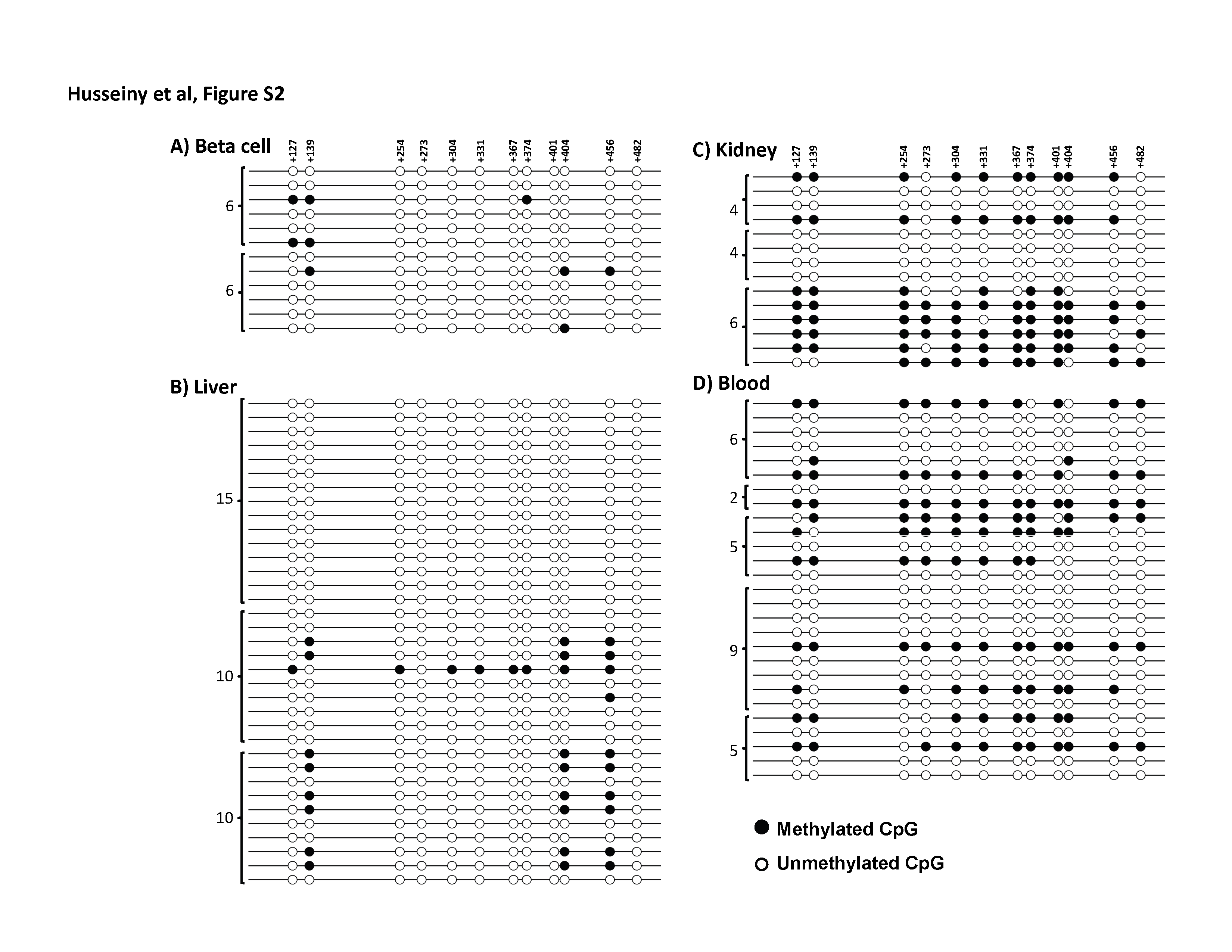

Supplement: Figure S2 — Methylation patterns of human INS exon2 obtained from different individuals. The human INS gene exon 2 was amplified with pairs of gene-specific primers for exon 2 (Table S1) using bisulfite-converted gDNA isolated from various tissues and different donors (brackets). Each PCR fragment was TA cloned into pCR2.1-TOPO vector and sequenced as described in Materials and Methods. PCR sequences were aligned with the expected sequence using the QUMA computer program (http://quma.cdb.riken.jp/). (A) Methylation pattern for beta cells resulted from 12 clones obtained from 2 individuals, (B) liver tissues resulted from 35 clones from 3 individuals, (C) kidney tissues resulted from14 clones from 3 individuals, and (D) blood tissues resulted from 27 clones from 5 individuals as indicated in (Table S2). (TIFF) [file pone.0094591.s002.tiff]

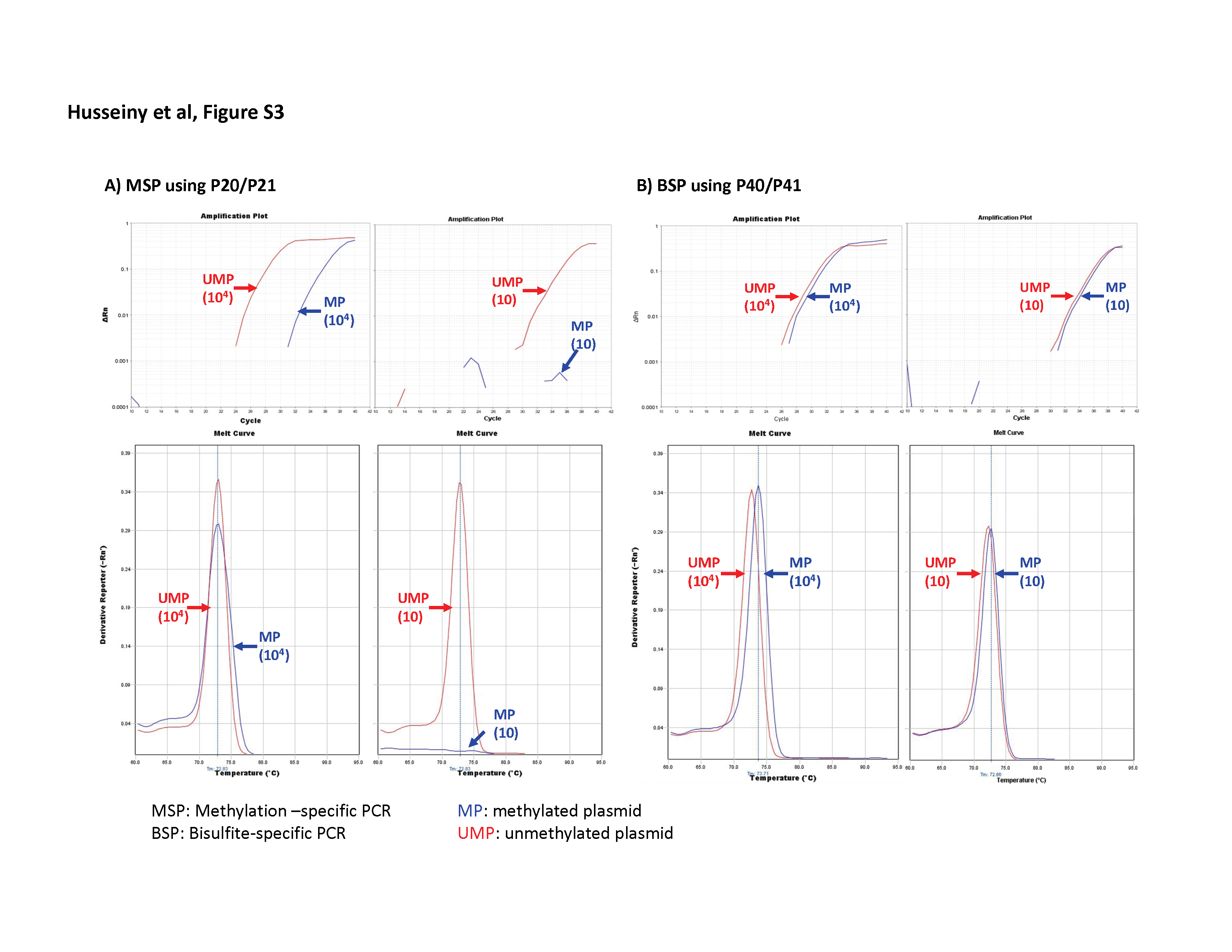

Supplement: Figure S3 — Differentiation between methylated and unmethylated sequences. The cloned human INS fragment in the pCR2.1 plasmid was methylated using M.SssI CpG methyltransferase, which methylates all cytosine residues within the double-stranded dinucleotide recognition sequence CpG. (A) Methylation-specific PCR (MSP) using primers P20/P21. (B) Bisulfite-specific PCR (BSP) using primers P40/P41. Amplification plots (upper) and melting curves (lower). The qMSP assay for 104 copies of the unmethylated sequence (red line) shows lower Cq values than 104 copies of the methylated sequence (blue line), and qMSP can detect 10 copies for unmethylated but not methylated sequence. In contrast the BSP assay shows the same Cq values for both unmethylated and methylated sequences at 104 and 10 copies. (TIFF) [file pone.0094591.s003.tiff]

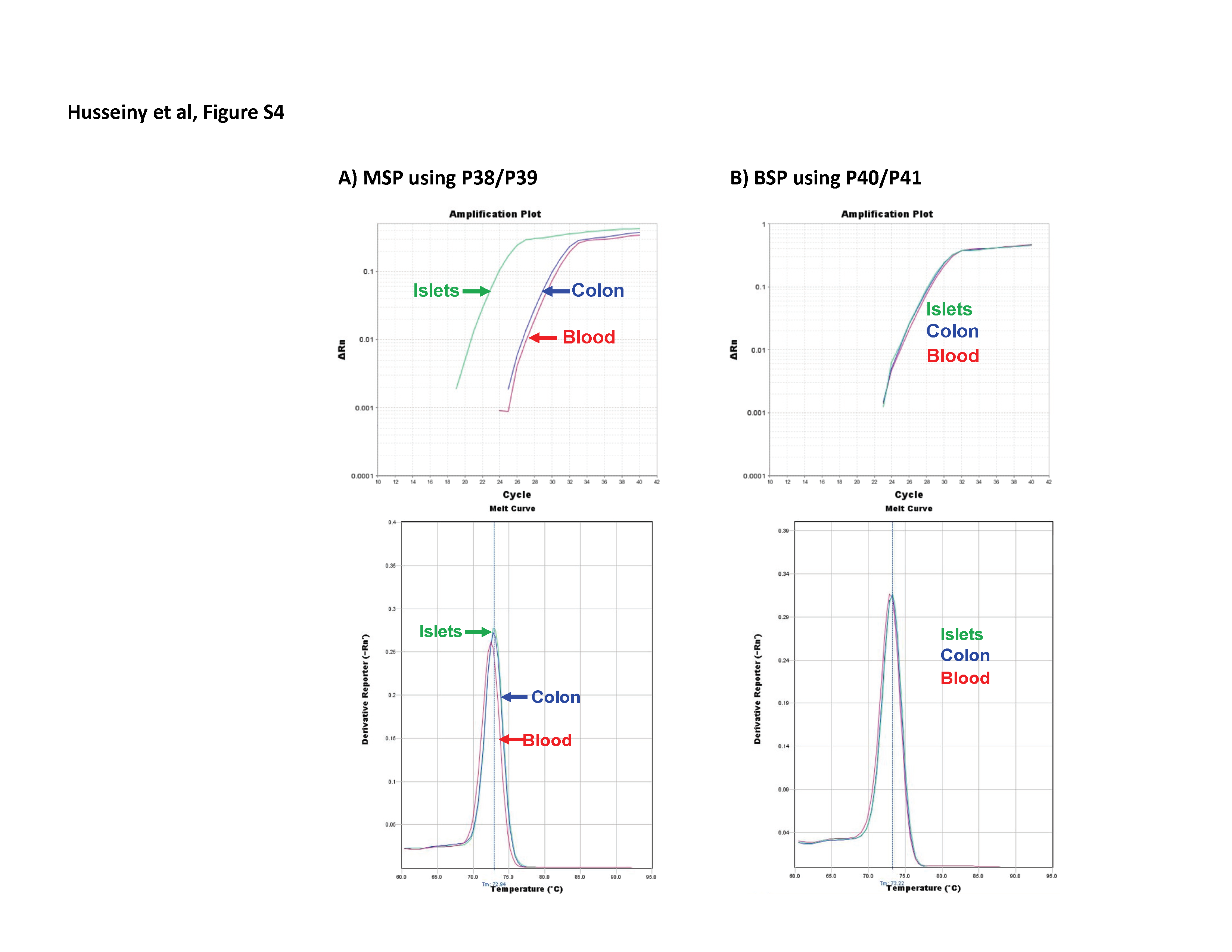

Supplement: Figure S4 — Melting curves from the 2nd step nested PCR reactions specific for beta cells. Bisulfite-converted gDNA obtained from human islets, blood and colon were used as a template for nested PCR. (A) First step MSP reaction using primers (P20/P21) followed by second step MSP reaction using primers P38/P39. (B) First step BSP reaction using primers (P40/P41) followed by second step BSP reaction using primers P40/P41. Amplification plots (upper) and melting curves (lower). The qMSP assay for human islets (green line) shows lower Cq values than human colon (blue line) and blood (red line) In contrast the qBSP assay shows the same Cq values for all tissues. (TIFF) [file pone.0094591.s004.tiff]
