# Supplementary material for: Tissue-Specific Methylation of Human Insulin Gene and PCR Assay for Monitoring Beta Cell Death
Source: PLoS One. 2014 Apr 10;9(4):e94591. doi: 10.1371/journal.pone.0094591 (PMC3983232; doi:10.1371/journal.pone.0094591)
Supplement: Table S1 — Oligonucleotides used in this study. (DOCX) [file pone.0094591.s005.docx]

**Table S1. Oligonucleotides used in this study.**

|  | **Designation** | **Sequence** |
| --- | --- | --- |
| **Primers for methylation mapping of human *INS* promoter** | | |
| 1 | HINSex2-For | 5’-GGTTTAGGATTTTAGGGTGGTT-3’ |
| 2 | HINSex2-Rev | 5’-CCCCCTTCTACCCATACTAAAT-3’ |
| **Primers for methylation mapping of human *INS* exon 2** | | |
| 1 | HuINS420-For | 5’- TGTGGGGATAGGGGTTTGGGGATAGTA-3’ |
| 2 | HuINS420-Rev | 5’- CCTCTTCTAATACAACCTATCCTAAAAAACTAAAAACTAC-3’ |
| **Primers for cloning human *INS*  gene** | | |
| 1 | H-INS-pro-For | 5’- TGTGGGGACAGGGGTCTGGGGACA-3’ |
| 2 | H-INS-exon2-Rev | 5’- AGCCTCCTGCCCCCTTCTGCCCAT-3’ |
| **Primers for qMSP** | | |
| P20 | H-Pro-Bisulf-For1 | 5’- ATAGGGGTGTGGGGATAGGGGTTTGGGGATAGTAGT -3’ |
| P21 | H-Pro-Bisulf-Rev1 | 5’- AACCCATCTCCCCTACCTCTCAACCCCTACCA -3’ |
| P38 | H-Pro-BS-For4 | 5’- TGGGTTTTTGGTTAAGATTTTAATGATTT -3’ |
| P39 | H-Pro-BS-Rev5 | 5’- CAACAAATAACTAAAAACTAAAACTACAATTTCCA -3’ |
| **Primers for qBSP** | | |
| P40 | MSP-For1 | 5’- ATAGGGGTGTGGGGATAGGGGTTTGGGGATAGTA-3’ |
| P41 | MSP-Rev1 | 5’- CAAAACCCATCTCCCCTACCTCTCAACCCCTAC -3’ |
