# Supplementary material for: Tissue-Specific Methylation of Human Insulin Gene and PCR Assay for Monitoring Beta Cell Death
Source: PLoS One. 2014 Apr 10;9(4):e94591. doi: 10.1371/journal.pone.0094591 (PMC3983232; doi:10.1371/journal.pone.0094591)
Supplement: Table S2 — Mapping of human insulin promoter and exon 2 regions. (DOCX) [file pone.0094591.s006.docx]

**Table S2: Mapping of human insulin promoter and exon 2 regions.**

|  | **Promoter** | | **Exon 2** | |
| --- | --- | --- | --- | --- |
| Organ tissues | Number of donors tissue | Number of clones | Number of donors tissue | Number of clones |
| Beta cells | 3 | 26 | 2 | 12 |
| Blood | 6 | 61 | 5 | 27 |
| Breast | 3 | 26 | 2 | 5 |
| Colon | 3 | 29 | 3 | 22 |
| Kidney | 3 | 25 | 3 | 14 |
| Liver | 3 | 24 | 3 | 35 |
| Lung | 3 | 24 | 3 | 19 |
| Spleen | 3 | 21 | 3 | 21 |
| Stomach | 3 | 20 | 3 | 13 |
